# Fatigue in radiology: a fertile area for future research

**DOI:** 10.1259/bjr.20190043

**Published:** 2019-05-13

**Authors:** Sian Taylor-Phillips, Chris Stinton

**Affiliations:** 1 Warwick Medical School, The University of Warwick, Coventry, England

## Abstract

Fatigue in radiologists may be responsible for a large number of medical errors. This review describes the latest research on fatigue in radiology. This includes measurement methods, and recent evidence on how fatigue affects accuracy in laboratory test conditions and in clinical practice. The extensive opportunities for future research in the area are explored, including testing interventions to reduce fatigue-related error, and further understanding of which fatigue measures correlate with errors. Finally we explore the possibility of answering these questions using large population-based observational studies and pragmatic integrated randomised controlled trials.

## Introduction

Fatigue in medical professionals is detrimental to wellbeing and morale, and may result in medical errors. In this review, we focus on the effect of fatigue on medical errors by radiologists. There may be more than 11,000 preventable hospital deaths in English NHS acute hospitals each year of which diagnostic error contributes to over eight thousand.^1^ This is a conservative estimate, with previous estimates serious disability or death caused by healthcare interventions in as many as 60 000 to 255 000 NHS patients.^2^ The proportion of these which can be attributed to fatigue is not known. However we do know that more radiological errors occur towards the end of a shift then at the beginning, and that longer shifts in medicine are associated with higher rates of medical errors, and personal injury.^3–5^ The problems are exacerbated because the workload of radiological services is increasing dramatically (by an estimated 10–12% per year) and this is occurring without a concomitant increase in the number of staff.^6^ While the potential harm from fatigue in radiology is very large, there is surprisingly little research in the area. In this review we examine the different types and classifications of fatigue, the current evidence base using laboratory test sets and in real world observational studies and trials, and discuss future research directions.

## Measurement of Fatigue

Fatigue is commonly described as feelings of weakness, lack of energy, and a desire to rest, and is associated with impairments in the ability to function.^7,8^ It is typically divided into two related subtypes: physical and mental fatigue. Physical fatigue is a deterioration in the muscles’ ability to create or sustain force, which results in difficulties in controlling and co-ordinating muscles, while mental fatigue is a reduction in the ability to perform mental tasks.^9^ In relation to radiology there is further subset: ocular fatigue related to vision. Measurement of fatigue can be objective or subjective, and each measurement method may cover one or more of these categories.

A range of tools have been developed to measure fatigue. It is of note that some measures used to examine fatigue were designed to measure tiredness. It much of the literature, fatigue and tiredness (or ‘sleepiness) and used interchangeably. Several reviews have examined these tools.^8,10,11^ The majority of these rely of subjective assessment in the form of self-reported rating scales or checklists. Shahid and colleagues described both short-term (*e.g.* Karolinska Sleepiness Scale,^12^ Stanford Sleepiness Scale^13^ and global measures of sleepiness (*e.g.* Epworth Sleepiness Scale Scale,^14^ Sleep Wake Activity Inventory.^15^^8^ Krupinski and Reiner reported on several additional measures, including the Pittsburgh Sleep Quality Index^16^ and the Swedish Occupational Fatigue Inventory (SOFI).^10,17^

Objective measures of fatigue have also been described. For example, the Maintenance of Wakefulness Test (which examines alertness during the day) in which participants sit in a dimly lit room and are instructed to remain awake; the Psychomotor Vigilance Task (a test of sustained attention, during which participants are instructed to respond to stimuli by press a button – the test measures the number of responses/failures to respond); and the Continuous Performance Test (another measure of sustained attention, during which participants are presented with unstimulating tasks and are instructed to respond to certain stimuli while ignoring others). Krupinski, Reiner, Waite and colleagues have described alternative approaches to the measurement of fatigue, such as monitoring of blood pressure, galvanic skin responses and heart rate.^10,11^ They also highlight the importance of visual fatigue in medical imaging. Interpreting medical images is a demanding and repetitive visual search task, and errors (false-negatives) are relatively common.^18^ Aside from self-reported measures, the main approaches to examining visual fatigue are through the assessment of accommodation (the adjustment of the curvature of the lens of the eye to change focus between objects in the distance and up close) and vergence (obtaining/maintaining binocular vision through the movement of eyes in opposite directions). Accommodation and vergence decline with fatigue.^10,11^ Others suggest critical fusion frequency (the frequency at which a flickering light is viewed as stable) and eye blink rate as objective measures of visual fatigue.^19,20^ Few of these measures have been employed in research on fatigue in medical imaging.

While there is reasonable consensus among researchers about the range of objective and subjective tools which are suitable to measure fatigue, there is very little research which links scores on these measures to clinically relevant outcomes from radiology, such as detection rates or error rates. Without knowing the relationship of these fatigue measures to clinical outcomes, studies using these measures are less likely to influence clinical practice. While it is important to minimise fatigue for the wellbeing of radiologists, understanding how fatigue can result in missing clinically relevant abnormalities may be more influential. Therefore, patterns of test accuracy, either measured continuously over the course of a radiology reading session or over the whole radiology work day, are often used as an alternative objective measure of fatigue. The psychology literature contains an abundance of studies examining the accuracy of novices at search and vigilance tasks typically lasting up to an hour.^21^ This field of research stemmed from an observation that sensitivity of radar operators to detect enemy aircraft or ships on radar screens decreased with time on task, called the vigilance decrement.^22^ The radiology reading task is considered a similar high intensity low signal salience task which may exhibit similar patterns,^23^ (although differences in expertise, task, and task importance may limit generalisability). Also well-established from the field of psychology is the prevalence effect, where radiologists sensitivity to detect abnormalities is lower when examining test sets with lower prevalence of abnormalities.^24^ This may be driven by radiologists expectations of the probability that a case is abnormal before looking at it, resulting in decreases in sensitivity with time on task in low prevalence radiology reading.^25^ There is also emerging research on visual adaptation to examining radiology images, with further exploration required to determine whether this impacts patterns of accuracy with time on task.^26^ There is a body of research about circadian rhythms (that being, biological processes that vary rhythmically over the course of, approximately, 24 h). In laboratory settings, time-related variations in performance have been observed for a range motor-, visual-, verbal-, memory-, and sensory tasks.^27^ In general, performance on tasks improves over the course of the day and declines during the night, albeit with fluctuations.^27^ There is some evidence of a dip in performance after lunch.^28^ Circadian rhythms have been hypothesised to apply to radiology accuracy, with some studies reporting that diagnostic accuracy is worse when the interpretation of images takes place later in the day than when it takes place earlier in the day.^29,30^ Other studies do not support this.^31,32^

## Recent research in the laboratory setting

Studies of the impact of fatigue on medical imaging are rare. Stec and colleagues conducted a systematic review looking for published research on the topic up to January 2017.^33^ They identified 27 relevant papers, fewer than half of which were primary studies (*n* = 10) or ‘other’ types of studies (*n* = 3). The remaining 14 papers were themselves reviews.

Krupinski et al conducted a series of experimental studies.^29,30,34,35^ In the first of these studies, detection of bone fractures was assessed in 40 readers (20 radiologists and 20 residents) who read a series of 60 cases, comprising 2–4 images, before (early condition) and after (late condition) a day of routine clinical practice.^29^ General fatigue was assessed using the SOFI, and visual fatigue (eye strain) was assessed using WAM-5500 Auto Refkeratometer (Grand Seiko, Hiroshima, Japan), which collects pupil diameter and refractive measurement. The authors found that subjective reports of fatigue and eye strain were significantly greater at the ‘late’ time point compared to the ‘early’ time point, and that diagnostic accuracy was significantly lower after a day of clinical practice than before (area under the curve early = 0.885, late = 0.852). No direct assessment was made between fatigue measurements and task performance.

In the second of their studies, 44 readers (22 attending radiologists and 22 radiology residents) sought to identify pulmonary nodules in CT of the chest.^30^ As in their previous study, performance was examined before and after a day of clinical practice. General fatigue was assessed using the SOFI, with dark vergence (convergence of the eyes in the absence of stimuli) used as a measure of eye strain. Similar results were observed to those of their earlier study^29^; diagnostic accuracy was significantly worse at the end of the day compared to the beginning of the day for the radiology residents (79% *vs* 75%). No such difference was observed for the attending radiologists. General and visual fatigue were not consistently higher in the late group. Degree of fatigue (*i.e.* score on the fatigue inventory or degree of visual strain) and task performance was not directly assessed.

In the third study, 20 radiologists examined computer tomography of multiple injuries after they had completed 8 h of clinical work (the so-called ‘fatigued’ group). Fatigue was measured as per the study of Krupinski et al.^29^ The fatigued participants were match to 20 non-fatigued participants who had taken part in previous studies by the same research group.^29,30^ For major fractures, there was no difference in detection between the fatigued and non-fatigued group (ROC AUC 0.945 *vs* 0.944, respectively). Again, no direct assessment was conducted between levels of fatigue and task performance. These studies provide some indication that radiologist expertise and experience may affect amount of fatigue experienced, and/or moderate the relationship between fatigue and accuracy, which merits further investigation.

Finally, Hanna et al examined the effect of overnight shifts on diagnostic accuracy and fatigue in 12 radiologists (five faculty members and seven radiology residents).^35^ The participants viewed bone radiographs during two conditions, once during the day (non-fatigued condition) and once in the morning after an overnight shift (fatigued condition). They also completed the SOFI. There were significant differences between the two conditions: there were higher fatigue scores on each of the domains of the SOFI, and lower diagnostic accuracy (0.806 *vs* 0.926) after the shift.

The results of these studies is supported by the review of Stec et al, which concluded that visual fatigue is relative common in radiologists (approximately 35% reported eye strain), and that visual and physical fatigue are associated with worse diagnostic accuracy (as measured by performance during extended periods of time).^33^

## Recent research in clinical practice

While the majority of evidence on the effect of fatigue on performance in medical imaging has been derived from experimental studies, a small number of studies have taken place in clinical practice. This is particularly valuable for understanding the importance of fatigue in the ‘real world’ as prior evidence has suggested variable associations between performance on experimental tasks and that observed in imaging in clinical practice.^36,37^

Ruutiainen and colleagues compared discrepancies between the preliminary and final interpretation of 8062 medical images of 10 radiology residents who were working long hours (more than 10 consecutive hours). They found that there was a significantly higher rate of major discrepancies (which potentially had an impact on patients) in reports from the final 2 h of the residents’ shifts compared to earlier shifts (2% compared to 1%).

Hanna et al retrospectively analysed the effects of shift length, schedule (regularly, holiday, weekend, extra, backup), and volume of workload on the accuracy of interpretation in a large sample (*n* = 2,922,377) of radiologic examinations covering a broad range of medical specialties.^5^ They found that both longer shifts and higher volumes of work (which are linked to fatigue) were associated with a greater number of major discrepancies in the interpretation of images. Further, a greater number of major discrepancies occurred during the latter part of shifts.

To the best of our knowledge, there has only been one prospective study of fatigue in medical imaging, the Changing case Order to Optimise patterns of Performance in Screening (CO-OPS) trial. This was a large randomised controlled trial that included data from over one million women. Taylor-Phillips and colleagues examined the impact of the vigilance decrement on the performance of pairs of readers (radiologists, radiography advanced practitioners, and breast clinicians) who were evaluating mammograms in the English breast cancer screening programme.^38,39^ The reader pairs interpreted batches of approximately 35 mammograms in succession. For half of the pairs, the readers reviewed the batches in the same order (the control group), and for the other half of the pairs one reader read the batches forward, and the other read the batches backwards (the intervention group). If the vigilance decrement was in evidence, the cancer detection rate should decrease with time on task in the control group (as by the end of the batch both readers would be in a low vigilance state) but not in the intervention group (as the readers will be in low vigilance states at different points in the batch). However, no difference was observed in cancer detection rates between the two groups (OR, 1.01; 95% CI, 0.97–1.06), indicating no vigilance decrement. Interestingly, recall rate decreased over time, suggesting that reader performance in terms of positive predictive value actually increased with time on task.

Further analysis of a subset of the CO-OPS dataset has been reported by Stinton et al.^38^ They examined recall and cancer detection rates by readers over the course of the day, with data were divided into three equal time periods, based on when the interpretations of mammograms were carried out: 9am to 4:59pm, 5pm to 12:59pm, and 1am to 8:59am. Variability in both recall and cancer detection rates were observed over the course of the day, and multilevel logistic regression indicating that females whose mammograms were interpreted between 5pm and 12:59pm were 1.07 (95% credible interval 1.03–1.11) times more likely to be recalled than females whose mammograms were read during each of the other time periods (*p* < 0.001). There was no significant association between time period and cancer detection rate. These patterns were observed in analyses with (1) all readers included, and (2) excluding readers who didn’t work during the evening.

## Future Research Opportunities

There is good evidence that fatigue is a serious issue affecting error rates across medicine including radiology. Future research is required to understand which interventions can reduce fatigue in radiologists, and in turn reduce the consequent medical errors. Further, there are many subjective and objective measures of fatigue, but research is required to identify which ones are associated with fatigue-related medical errors. Such information could be used to select fatigue measurements which would identify states of fatigue that may lead to medical errors in real-time. Elsewhere in radiology, technological advances associated with artificial intelligence may affect several elements of the radiology task including those driving fatigue, and such associations require investigation. Finally, the advent of big data provides an opportunity to answer these research questions using methods that were not previously widely available, in large-scale observational and integrated pragmatic trial studies.

There is a scarcity of research investigating the effectiveness of interventions to reduce fatigue and fatigue-related errors in radiology. The largest study was a randomised controlled trial of an intervention to change case order in mammography screening detailed above.^39^ This study found that the intervention was not effective. The effect of other potential interventions to reduce fatigue in a single reading session, such as break scheduling, interruptions, caffeine, workstation design and ambient lighting, are yet to be robustly tested in a real-world setting. Perhaps the greater opportunity is in investigating fatigue towards the end of a long shift, which may be greater than that which develops within a single radiology session. Interventions in this context may be more organisational such as scheduling of tasks within a shift so that safety critical tasks are towards the beginning, or changes to shift length, shift times, or staffing levels. All such interventions should be designed to support radiologists in managing their own fatigue, rather than taking a top-down mandated approach, and so necessarily study designs to investigate these must be pragmatic and include adaptation to the local context.

We do not know which fatigue measurements are related to increases in rates of medical errors. Large studies are required employing several simple fatigue measures in the same radiologists at the same time, and relating these to real-world error rates. These data would inform which fatigue measures can be used as a proxy for fatigued states that cause medical errors. Then the same measures could be used as proxy outcomes in research studies, and as useful feedback mechanisms in clinical practice.

The advent of artificial intelligence examining radiological images is not new, it is akin to computer aided detection, which has been implemented for many years with varying levels of success. What is new is developments in data storage, linkage and artificial intelligence are resulting in a new generation of automated image reading tools. The accuracy of these is not the focus of this review, but accuracy appears to be dependent on access to large numbers of images with known ground truth, which are becoming increasingly available. What is important from the perspective of radiologist fatigue, is how these developments interact with the radiologist. Fatigue research has shown that in some circumstances specificity and positive predictive value improve as the reading session progresses,^40^ which may explain the mechanism through which batch reading improves specificity.^41^ Building on this human factors and fatigue research we can postulate that if artificial intelligence was used to sift out the straightforward normal cases then this would increase case difficulty read by radiologists, which may increase fatigue but also increase prevalence and therefore sensitivity.^23,24^ If artificial intelligence were implemented to highlight suspicious radiological areas then it may interrupt the reading session with false positive prompts, and negatively affect specificity, in a similar manner to its predecessor, computer-aided detection in breast screening.^42,43^

The challenge in measuring radiologist fatigue is one shared by researchers measuring all elements of radiologists’ performance and accuracy, that of statistical power. We want to know which equipment, workstations, working hours, conditions and tests enable radiologists to be as accurate as possible. We want the outcomes of our research studies to be clinically meaningful, for example measuring numbers of clinically significant missed cases/abnormalities. This requires either test sets enriched with unrealistic numbers and types of abnormalities, or very large studies. As there is reasonable evidence that radiologists’ behaviour examining enriched test sets is not generalisable to clinical practice,^36,37^ the future appears to lie in very large studies.

The era of ‘big data’ provides great potential and risk for research. Computerised patient records and images on a large scale provide fantastic research opportunities that are only beginning to be realised. Breast cancer screening in England is an example at the forefront of this. This screening programme has one single computer programme to manage patient records, so recording is relatively uniform across the country. This computer programme has regular data linkage to and from the national cancer registry which contains long term patient follow-up data. This has allowed a wealth of observational research, and more recently changes to the software to integrate randomisation. This internal randomisation, and follow up to clinically relevant outcomes allows us to automate the running of randomised controlled trials such as the CO-OPS trial of one million women^39^ and the age extension trial of six million.^44^ Such automation allows much larger trials at significantly lower cost. Where possible such pragmatic integrated randomised controlled trials provide the least biased form of fatigue research available. Where randomisation is not possible or practical, for example comparing different reading room designs, observational research remains valuable.

In the transition to use of big data routinely in radiology research and audit there is a real risk of inappropriate analyses leading to incorrect conclusions. There is now a plethora of observational data available in radiology, in the images themselves and the associated patient data. Observational study designs have many confounders and caution is required in drawing conclusions from such designs. The increased availability of large sample sizes could lead to increased publication bias through publication only when results are ‘interesting’. For example in the UK an observational analysis of increased death rates of patients admitted to hospitals at weekend was used as rationale for seven day working.^45,46^ However, a driving factor of the effect is increased severity of illness in those admitted at the weekend, with the authors themselves stating that “to assume that they [extra deaths at weekend] are avoidable would be rash and misleading”. This is one of the more prominent examples of the difficulties of ascribing cause and effect when only observational data are available. Research methods training should become more widely available to those who audit and analyse such data, to ensure analysis takes account of confounders and to prevent incorrect inference. In fatigue research, particular attention should be paid to individual differences between radiologists, for example through including radiologists in analyses as a random effect.

The success of future research in fatigue will depend heavily on the culture in radiology work environments. An individual radiologist’s performance data have the potential to be a very powerful tool for constantly improving performance, if communicated accurately in a positive learning environment. Similarly, research can be completely anonymised with respect to both patients and radiologists, allowing large research studies with lower perceived risk. If such data are used in an adversarial manner, as performance management, in a blame culture, or even in misconduct lawsuits then big data could be perceived as big brother. This would limit the willingness of radiologists to engage with big data in an open manner conducive to learning and improvement.

## Conclusion

There is clear evidence that fatigue, particularly towards the end of a long shift, contributes to serious medical errors, and increases the risk of missing abnormalities on imaging. There is a research need to investigate which interventions could reduce fatigue-related errors, and which measures of fatigue are strong early indicators of a fatigued state that may lead to medical errors. The advent of big data in healthcare provides an opportunity to undertake large studies to address these questions, but attention must be paid to addressing confounding.
